# A Modified Rabbit Ulna Defect Model for Evaluating Periosteal Substitutes in Bone Engineering: A Pilot Study

**DOI:** 10.3389/fbioe.2014.00080

**Published:** 2015-01-06

**Authors:** Rania M. El Backly, Danilo Chiapale, Anita Muraglia, Giuliana Tromba, Chiara Ottonello, Federico Santolini, Ranieri Cancedda, Maddalena Mastrogiacomo

**Affiliations:** ^1^DIMES, University of Genova, Genova, Italy; ^2^IRCCS AOU San Martino–IST Istituto Nazionale per la Ricerca sul Cancro, Genova, Italy; ^3^Faculty of Dentistry, Alexandria University, Alexandria, Egypt; ^4^Biorigen S.R.L., Genova, Italy; ^5^Sincrotrone Trieste S.C.P.A., Trieste, Italy

**Keywords:** periosteal substitute, platelet-rich plasma, critical size defect, e-PTFE, bone regeneration

## Abstract

The present work defines a modified critical size rabbit ulna defect model for bone regeneration in which a non-resorbable barrier membrane was used to separate the radius from the ulna to create a valid model for evaluation of tissue-engineered periosteal substitutes. Eight rabbits divided into two groups were used. Critical defects (15 mm) were made in the ulna completely eliminating periosteum. For group I, defects were filled with a nanohydroxyapatite poly(ester urethane) scaffold soaked in PBS and left as such (group Ia) or wrapped with a tissue-engineered periosteal substitute (group Ib). For group II, an expanded-polytetrafluoroethylene (e-PTFE) (GORE-TEX^®^) membrane was inserted around the radius then the defects received either scaffold alone (group IIa) or scaffold wrapped with periosteal substitute (group IIb). Animals were euthanized after 12–16 weeks, and bone regeneration was evaluated by radiography, computed microtomography (μCT), and histology. In the first group, we observed formation of radio-ulnar synostosis irrespective of the treatment. This was completely eliminated upon placement of the e-PTFE (GORE-TEX^®^) membrane in the second group of animals. In conclusion, modification of the model using a non-resorbable e-PTFE membrane to isolate the ulna from the radius was a valuable addition allowing for objective evaluation of the tissue-engineered periosteal substitute.

## Introduction

Metaphyseal long bone defects in animal models are commonly used to evaluate bone repair/regeneration since a high proportion of fracture injuries in human beings occur in long bones. These animal models often employ the creation of critical size defects (ASTM Standard F2721-09, 2009). Of these, the rabbit ulna or radius defect (between 10 and 20 mm) is often used as it is of relatively low cost and does not require any fixation owing to the support offered by the adjacent bone (Horner et al., 2010).

Several studies have used this model to evaluate the efficacy of tissue-engineered constructs to enhance bone repair, including the use of allogenic peripheral blood-derived mesenchymal stem cells associated with ceramic scaffolds (Wan et al., 2006), adipose-derived stromal vascular fraction (SVF) cells (Kim et al., 2012), novel alloy-based scaffolds (Smith et al., 2012), tri-phasic release of rhBMP-2 (Bae et al., 2011), and platelet-rich plasma (Kasten et al., 2008). All of these studies showed a clear fusion between the radius and the ulna at the sites of defect formation as a biologic response from cells from the surrounding tissues, thus masking the contribution of the implanted construct (Kasten et al., 2008). Periosteal remnants from the proximal and distal ends of the defect, in addition to the membrana interossea found between the two bones, may well be responsible for the synostosis or fusion between the radius and the ulna of the rabbit in these bone regeneration studies (Bodde et al., 2008).

Live periosteum is essential for autogenous bone graft healing and remodeling. It is living cells in the periosteum and endosteum as well as stromal cells that are responsible for 90% of early osteogenesis while free marrow cells and osteocytes in the live autograft have little or no contribution (Zhang et al., 2008). Targeting periosteum-mediated bone repair offers the benefits of healing with natural bone structure, optimal bone integrity, appropriate vascularization, and minimal ectopic calcification (Fan et al., 2010). Indeed, recent studies, including one from our laboratory, have attempted to mimic the periosteal response to enhance bone regeneration by engineering periosteal substitutes (Schonmeyr et al., 2009; Zou et al., 2009;Fan et al., 2010; Ma et al., 2011;Zhao et al., 2011). In order to precisely estimate the contribution of an engineered periosteal substitute in a critical size defect, the defect must be completely rid of all remaining periosteum and cell elements (Reichert et al., 2009). In fact, the absence of a clear separation between the two bones makes it difficult to evaluate the real effects of engineered periosteal substitutes.

Hence, we propose the use of a non-resorbable barrier membrane to separate the radius from the ulna without interfering with the dynamics of healing as a valid modification of the rabbit ulna defect model for periosteal engineering studies. Expanded-polytetrafluoroethylene (e-PTFE) membranes (GORE-TEX^®^) have long been used to minimize surgical adhesions (Dessanti et al., 2000). These membranes are non-resorbable, show no immunoreactivity, and function as efficient protective barriers (Minale et al., 1987; Loebe et al., 1993; Jacobs et al., 1996; Kaushal et al., 2011; Kumar et al., 2011). Expanded PTFE surgical membranes are also routinely used for Guided Tissue and Bone Regeneration (GTR and GBR) (Schliephake et al., 2000; Zybutz et al., 2000; Simion et al., 2007; Lindfors et al., 2010; Retzepi and Donos, 2010).

We have recently engineered and characterized a periosteal substitute comprising a platelet-rich plasma gel membrane entrapping autologous bone marrow mesenchymal stem cells (BMSC) as a periosteal substitute for bone engineering having enhanced angiogenic and osteogenic properties (Elbackly et al., 2012). The rationale of the current study was to modify the conventional rabbit ulna defect model by first adding an e-PTFE membrane (GORE-TEX^®^) to isolate the radius from the ulna, which would allow a precise evaluation of the effect of engineered periosteal substitutes without the influence of cellular remnants from the radius.

## Materials and Methods

### Culture of rabbit BMSC and preparation of platelet-rich plasma/BMSC periosteal substitutes

Bone marrow mesenchymal stem cells were cultured from iliac crest marrow aspirates of four male white New Zealand rabbits weighing 2.5–2.7 kg as mentioned before (Elbackly et al., 2012). Briefly, mononuclear cells were plated and cells were cultured in Coon’s modified Ham’s F-12 medium containing: 2 mM glutamine, 100 U/ml penicillin, 100 μg/ml streptomycin (Sigma Chemical Co., St. Louis, MO, USA), and 10% fetal bovine serum (FBS) (Life Technologies, Invitrogen^®^) in the presence of 1 ng/ml human recombinant Fibroblast Growth Factor 2 (FGF2) (Peprotech^®^, London, UK).

PRP and platelet poor plasma (PPP) were prepared from a pool of whole blood obtained from five rabbits as before (Elbackly et al., 2012). Briefly, the blood was centrifuged at 209g for 16 min then the plasma supernatant with the underlying buffy coat was collected. This was then centrifuged at 1500g for 12 min to sediment the platelets and the clear supernatant phase was collected as PPP. The platelet pellet was re-suspended in PPP to obtain PRP with a final concentration of 3 × 10^6^ platelets/μl. PRP and PPP were stored at −20°C until use.

PRP/BMSC gel membranes were prepared using: PRP, PPP, bovine thrombin, and Ca gluconate in an 8:1:1:1 ratio, respectively. Autologous first passage rabbit BMSC were trypsinized, and re-suspended in a mixture of 1.6 ml PRP and 200 μl PPP. Each gel membrane contained 6 × 10^6^ BMSC. A mixture of 200 μl Ca gluconate and 200 μl thrombin (final concentration: 9 IU/ml) (Multifibren U, Siemens, Germany) was added. The gel membrane was then left to jellify at 37°C in 5% CO_2_ for a minimum of 1 h until surgery.

Before surgery, the PRP/BMSC gel membrane was wrapped around a nanohydroxyapatite/poly (ester urethane) (nHA/PU) cylindrical scaffold (provided by the AO Research Institute, Davos, Switzerland) (Elbackly et al., 2012), which had been previously soaked overnight in PRP. This scaffold has a diameter of 4 mm and height of 15 mm. It carries the advantage of the combined visco-elastic properties of PU with the increased scaffold stiffness and osteoconductive nature offered by nHA. These scaffolds can also support ectopic bone formation (data not shown). For the control groups, the scaffold was soaked in PBS.

### Animals and surgical procedure

All experimental animal procedures were carried out in the IRCCS AOU San Martino–IST Animal Facility, in respect of the national current regulations regarding the protection of animals used for scientific purpose (*D.lgsvo 27/01/1992, no. 116)*. Research protocols have been evaluated and approved by the IRCCS AOU San Martino–IST *Ethical Committee for animal experimentation (CSEA)* as Animal use project no. 334 communicated to The Italian Ministry of Health, having regard to the article 7 of the D.lgs 116/92.

Eight adult male rabbits were used and they were divided into two main groups according to the absence (group I) or presence (group II) of a GORE-TEX^®^ isolating membrane around the radius. Each group was further sub-divided into two groups according to the treatment of the defect: group (a): defect filled with scaffold soaked in PBS (*n* = 2) and group (b): defect filled with scaffold wrapped with PRP/BMSC gel membrane (*n* = 2).

**Figure 1:**
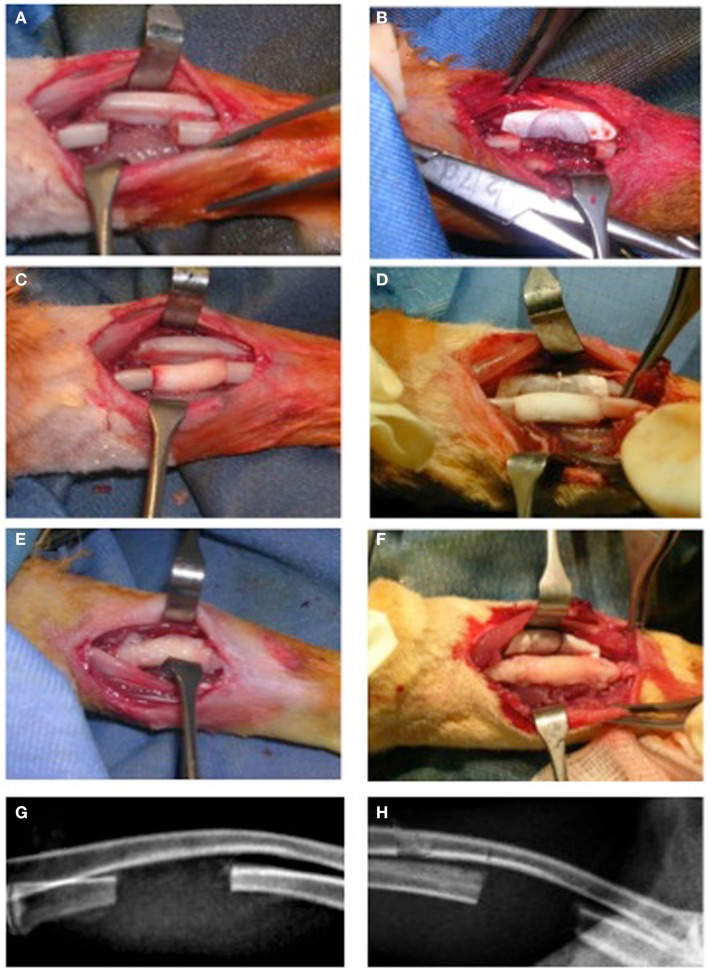
**Surgical creation of critical size segmental bone defect (15 mm) in the rabbit ulna**. **(A,C,E,G)** The traditional approach (NO GORE-TEX GROUP) and **(B,D,F,H)** modification of the technique by insertion of a non-resorbabale e-PTFE (GORE-TEX) membrane wrapped around the radius to separate it from the ulna (GORE-TEX GROUP). **(A)** The bone defect with periosteum completely removed from cut bony edges as well as from the adjacent radial surface. **(B)** The GORE-TEX membrane wrapped and sutured around the radius in the defect zone. **(C)** and **(D)** Defects filled with poly (ester urethane) scaffold with PBS (groups Ia and IIa, respectively). **(E)** and **(F)** Defects filled with the scaffold wrapped with the periosteal substitute (PRP/rBMSC membrane) with 2 mm of this membrane covering denuded bony edges at both medial and distal ends of the defects (groups Ib and IIb, respectively). **(G)** and **(H)** 6-day post-operative radiographs for a representative rabbit from each group.

Rabbits were anesthetized using Diazepam (1 mg/kg, Hospira, Italy) followed by Ketamine HCL (35 mg/kg, Merial, Italy) and Xylazine HCL (5 mg/kg, Bio 98 Srl, Italy) in addition to a local injection of Naropin (ropivacaine HCL, 7.5 mg/ml) (2 mg/ml, AstraZeneca S.p.A., Italy). Briefly, an oscillating saw on a battery hand piece (Howmedica, GmbH, Germany) with copious saline irrigation was used to create a 15 mm defect in the rabbit ulna. The periosteum was resected with the bony segment as well as 3–5 mm from the proximal and distal ends of the cut bony stumps. The periosteum of the adjacent radial surface was also removed followed by irrigation to ensure maximum elimination of any periosteal tissue remnants (Figure 1A). Furthermore, for groups (IIa) and (IIb), a non-resorbable e-PTFE membrane (GORE-TEX^®^, regenerative membrane, W.L. Gore & Associates, Inc., USA) was placed around the radius at the site of the defect to avoid a possible periosteal reaction and formation of a radio-ulnar synostosis (Figure 1B). The scaffold was then gently compressed into the defect (Figures 1C,D). For the groups receiving the PRP/BMSC gel membrane, the edges of the membrane were adjusted to overlap the denuded bony stumps (Figures 1E,F). Subcutaneous tissue layers and skin were closed with vicryl 4/0 suture thread and 3/0 silk sutures, respectively.

### Radiographic assessment and computed microtomography (μCT) analysis

**Figure 2:**
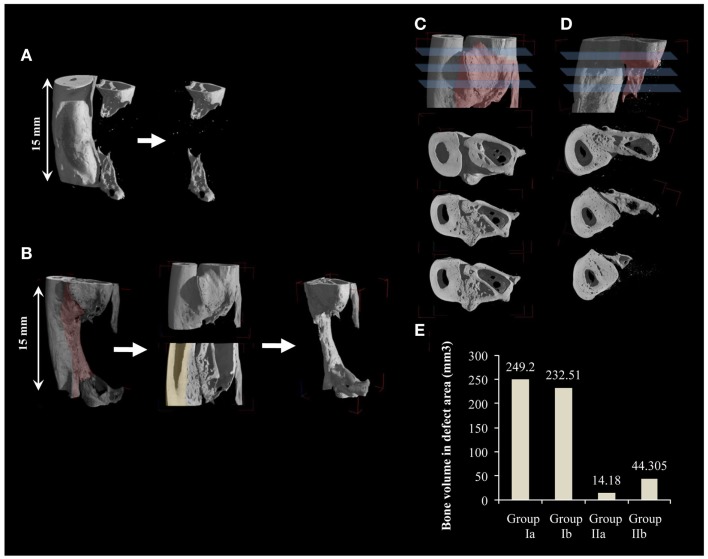
**Quantification of new bone volume via 3D μCT analysis**. **(A,B)** 3D μCT reconstructions for a sample from groups Ib and IIb, respectively. **(A)** Smooth radial surface shows where the margins of the created defect are delineated by the imprint of the GORE-TEX membrane. Quantification of bone in-growth for GORE-TEX groups was done by first identifying the defect area (15 mm) using the imprint of the GORE-TEX membrane then bone in-growth in the defect area was quantified. **(B)** Shaded area highlights the periosteal reaction. For the no GORE-TEX group, quantification was done by first taking, serial longitudinal slices throughout the sample. These were analyzed to detect the original cortical surface of the radius (shaded) by visualizing the change in bone architecture (from compact cortical plate of radius to highly trabecular new bone formed at the radio-ulnar junction due to extensive periosteal reaction). The area was then manually selected including the entire bone in-growth in the defect area (15 mm). This volume was then quantified. **(C)** and **(D)** cross sections at three different levels of one end of a defect from group Ib and IIb, respectively, showing in **(C)** complete fusion between newly regenerated bone in the defect area and the radius while in **(D)** new bone forms distinctly separate from the adjacent radius confirming the contribution of the periosteal-like substitute to the bone regeneration process. **(E)** Quantification of new bone volume in the defect area in mm^3^.

Radiographs were performed after 6 days (Figures 1G,H), and then monthly until euthanization. Transmission X-ray μCT was performed using a microfocus X-ray source with cone beam geometry at the TOMOLAB station (http://www.elettra.trieste.it/lightsources/labs-and-services/tomolab/tomolab.html) as previously mentioned (Elbackly et al., 2012). Three dimensional (3D) images were reconstructed from the series of 2D projections using the classical filtered back projection algorithm. VG Studio MAX software (Volume Graphics GmbH, Heidelberg, Germany) was used to produce 3D volume renderings (Figure 2). For the GORE-TEX group (II), identification of the defect area was done in the volume rendering of the scanned area (15 mm) by means of visual observation of the relative position of the GORE-TEX membrane denoted as the region of interest (ROI) (Figure 2A). Bone in-growth in the ROI was then isolated and quantified. For the NO GORE-TEX group, the ROI was identified based on visually observing longitudinal serial slices throughout the sample to identify the original dense structure of the radial cortical plates from the rarified structure of the newly regenerated bone (representing the synostosis + new bone regenerated in defect area). Bone in-growth was then quantified (Figure 2B). Bone volume (BV) regenerated in the defect area was calculated in mm^3^ all groups.

### Histological analysis

After euthanization, harvested rabbit ulnas were fixed in 3.7% paraformaldehyde and dehydrated in a graded series of ethanol (70, 90, and 95% absolute ethanol). Specimens were processed for undecalcified resin embedding. They were infiltrated with light-curing resin Technovit 7200 VLC (Kulzer, Wehrheim, Germany) and polymerized by the EXAKT 520 polymerizator system (EXAKT Technologies, OK, USA). Longitudinal sections were then cut and ground using the EXAKT 310 CP cutting and EXAKT 400 CS micro grinding units to a final thickness of 30–40 μm. Sections were then stained using Stevenel’s blue/Van Geison picro-fuchsin stain (SVG). Images of the sections were acquired using an Axiovert 200M microscope (Zeiss, Germany).

## Results

### Radiographic assessment and computed microtomography (μCT) analysis

**Figure 3:**
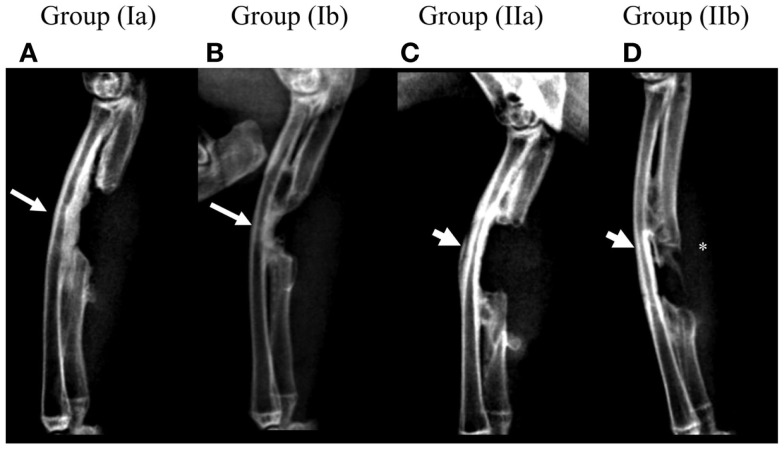
**Radiographic follow-up for one representative animal from each group at 3 months post-operatively**. **(A)** Group (Ia); **(B)** Group (Ib); **(C)** Group (IIa); **(D)** Group (IIb). Presence of a distinct periosteal reaction and radio-ulnar synostosis in the defect area can be seen in groups (Ia) and (Ib) (arrows) while it is absent in groups (IIa) and (IIb) (arrow heads). New bone forming in the defect area in group (IIb) (*) appears clearly distinct from the radial surface.

Post-operative radiographs for group (Ia) showed a thickening of the radial cortical plate facing the defect area with formation of radio-ulnar fusion while the defect area itself remained devoid of new bone formation (Figure 3A). Group (Ib) showed the formation of new bone in the defect area commencing from both proximal and distal ends of the defect. This new bone appeared fused with the radial cortical plate due to its thickening (Figure 3B). Group (IIa) defects showed no bone formation in the defect area or in the form of thickening of the radial cortical plate. Minimal radio-ulnar fusion could be seen only beyond the limits of the defect area isolated by the GORE-TEX membrane (Figure 3C). Group (IIb) showed the formation of a bridge of new bone attempting to traverse the defect area. This remained completely separate from the radial cortical plate by virtue of the GORE-TEX membrane (Figure 3D).

Post-mortem μCT images confirmed the radiographic evaluation. In the GORE-TEX group, particularly group (IIb) where new bone formed in the defect area, the radial surface appeared smooth with clear demarcation of the zone of the GORE-TEX membrane from the newly formed bone (Figure 2A). In the NO GORE-TEX group especially (Ib) (Figure 2B), excessive new bone apposition could be seen on the radial surface facing the defect area (shaded area) evidenced by the difference in bony architecture with the presence of new bone formation from the bony edges of the defect as well as new bone traversing the defect. Transverse sections in groups (Ib) and (IIb) (Figures 2C,D) showed that for group (Ib), all levels within the bone regenerated in the defect area showed complete fusion between the new bone formed and the radial surface. Whereas, for group (IIb), the new bone formed appeared clearly separate from the radial surface along the entire length of the zone in which the GORE-TEX membrane was placed. The extracted BV in the defect area was 249.2 mm^3^ for group (Ia), 232.51 mm^3^ for group (Ib), 14.18 mm^3^ for group (IIa), and 44.31 mm^3^ for group (IIb) (Figure 2E).

### Histological analysis

**Figure 4:**
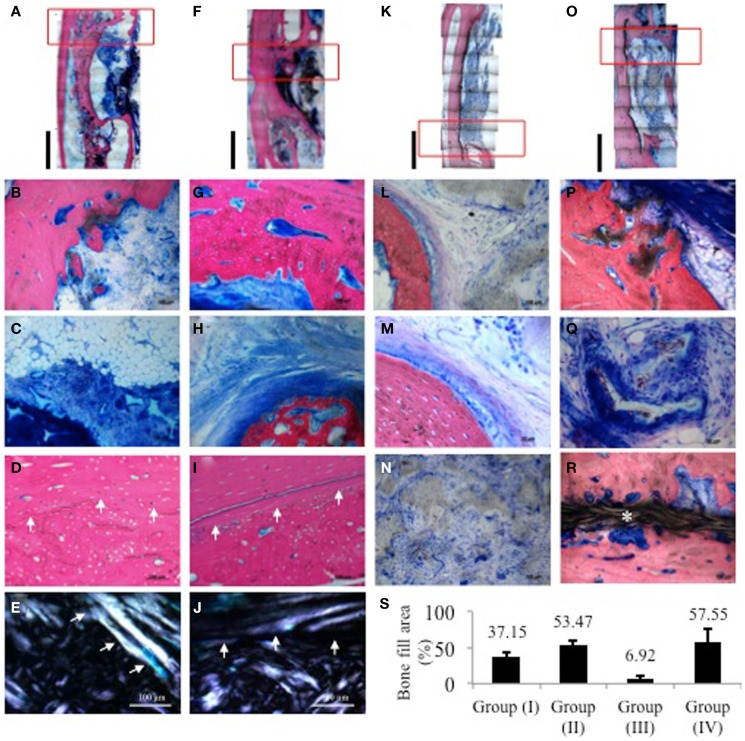
**Histological images of samples from one representative animal per group stained with SVG**. Red rectangles indicate the areas from which higher magnification images were taken. **(A,F,K,O)** Low power reconstructions of histological images of defects from group (Ia), (Ib), (IIa), and (IIb), respectively (Bar = 5 mm). **(B–E)** group (Ia); **(B)** border of defect with limited bone activity and isolated bone trabecule; **(C)** central area of defect showing accumulation of adipose tissue among highly cellular connective tissue infiltrating the remaining scaffold structure; **(D)** radio-ulnar synostosis zone showing clear transition between cortical bone of the radius and new bone deposited on its surface showing presence of remodeling osteons and micro-cracks; **(E)** same sample viewed under polarized light showing difference in collagen fiber orientation between original cortical bone and new bone deposited in the zone of synostosis. **(G–J)** Group (Ib); **(G)** border of defect showing substantial new bone deposition lined with a layer of osteoid matrix; **(H)** central part showing trabecule of woven bone attempting to bridge the remaining defect area embedded in a dense matrix of collagen fibers. Osteoblasts depositing osteoid matrix line the trabecule; **(I)** radio-ulnar synostosis zone further emphasized under polarized light in **(J)**. **(L–N)** group (IIa); **(L)** border of defect adjacent to remaining scaffold; **(M)** higher magnification of border showing mature lamellar bone and minimal new bone deposition; **(N)** cell infiltration in center of scaffold; **(P–R)** group (IIb); **(P)** new bone depositing on the scaffold with adjacent developing marrow; **(Q)** mineralizing collagen fibers in between and within the scaffold structure; **(R)** new bony bridge traversing the defect peripherally above the GORE-TEX membrane clearly separate from the radial surface. **(S)** Quantification of bone fill area% from histological sections. For each sample, three sections were analyzed where the distance between each section was approximately 200–300 μm.

Healing was uneventful, and there were no signs of inflammatory response in either of the groups. Reconstructions from low power histology images showed in group (Ia), minimal bone formation from the bony edges of the defect yet with bone deposited on the surface of the adjacent radius (Figure 4A). Higher magnification revealed some new bone deposition in the form of isolated bony islands close to the border (Figure 4B). Central areas showed accumulation of adipose tissue among highly cellular connective tissue infiltrating the remaining scaffold structure (Figure 4C). The zone of radial hypertrophy can be distinguished by a clear demarcation between the woven structure of the new bone apposed on the radius and the lamellar structure of the cortical plate of the radius (Figure 4D). Developing osteons and micro-cracks can be seen in the new bone. This demarcation is further accentuated under polarized light due to the difference in collagen fiber orientation between the woven and lamellar bone (Figure 4E).

In group (Ib), the defect area appears much smaller owing to new bone formation attempting to bridge the defect (Figure 4F). In this group, hypertrophy of the radius appears much thicker with fusion between the radius and newly deposited bone resulting in further reduction of the size of the defect as compared to group (Ia). Magnification at one end of the defect shows active new bone deposition lined with a well defined layer of osteoid matrix (Figure 4G). Centrally, an island of *de novo* bone can be seen in a dense collagenous matrix (Figure 4H). The zone of fusion is again distinguishable by the difference in the bone structure between the radial cortex and the new bone apposed on its surface under both transmitted and polarized light (Figures 4I,J). In both groups (Ia) and (Ib), new bone formation appears to have begun from either end of the defect as well as from the adjacent radial surface.

In group (IIa), the size of the defect zone remained to a great extent unchanged with little formation of new bone as shown in the low power reconstruction of the defect (Figure 4K). There is almost no bone remodeling evident. The scaffold structure shows some signs of degradation, yet it is still grossly present throughout most of the defect (Figure 4L). The bone surface shows limited osseous activity with attempts at new bone formation evidenced by a thin layer of osteoid matrix lined by osteoblasts (Figure 4M). The GORE-TEX membrane appears intact and well adapted to the radial surface clearly isolating the defect area (Figure 4N).

In group (IIb) defects, a substantial amount of new bone has formed in the defect area starting peripherally from either end (Figure 4O). The new bone appears to be mapping out the scaffold structure and shows marrow development (Figure 4P). Dense mineralizing collagen bundles are clear at the edges of the newly forming bone intertwined with the degrading scaffold. Mineralized deposits are also seen within the remaining scaffold pores (Figure 4Q). The GORE-TEX membrane is interposed between the cortical bones of the radius and clearly distinguishes it from the new bony bridge, which has formed guided by the PRP/BMSC gel membrane (Figure 4R).

Histomorphometric quantification of the percentage of bone filling the defect area showed that for groups (Ia) and (Ib), the percentage of bone fill was 37.15 and 53.47%, respectively, whereas it was 6.92 and 57.55% for groups (IIa) and (IIb), respectively (Figure 4S).

## Discussion

In the present work, we showed that a non-resorbable GORE-TEX membrane could act as an effective barrier between the radius and a rabbit ulnar defect preventing hypertrophy of the radial cortical bone and synostosis. Although the rabbit segmental bone defect in the radius or ulna is commonly used in bone regeneration studies, the fibro-osseous union between the two bones allowing these defects not to require fixation is also the reason for the frequent fusion between them seen in most studies conducted using this model (Wan et al., 2006; Bodde et al., 2008; Zhao et al., 2011). When evaluating a periosteal substitute for bone engineering, this phenomenon becomes of major importance especially since many studies have not defined adjacent bone hypertrophy or growth as defect bridging.

For this reason, removal of the periosteum from both ends of the defect in addition to the intervening interosseous membrane has been suggested (Bodde et al., 2008) to eliminate possible progenitor cell sources that may influence result interpretation. However, this may not be sufficient as scratching of the bone surface maybe sufficient to initiate a biologic reaction leading to hypertrophy. This reaction may be due to activation of periosteal cambial cells resulting in callus matrix synthesis (Simon et al., 2003; Bodde et al., 2008; Cho et al., 2011). This inherent nature of periosteal activation has recently been utilized for a novel osteogenesis technique based on GBR. Gradual periosteal elevation and creation of a space overlying the bone surface can lead to stimulation of bone formation in the space. By adding a barrier membrane to prevent invasion of this space by non-osteogenic cells, this technique can be used to favor endogenous bone repair mediated by the periosteum (Zakaria et al., 2012).

Indeed, in the present work, even though for groups (Ia) and (Ib) the periosteum from the bony stumps as well as from the adjacent radius was removed, a periosteal reaction occurred even in the control group and more so in the group that received the periosteal substitute. This resulted in an over-estimation of BV in μCT images and a failure to perceive the difference between the two groups. Although histological evaluation in groups (Ia) and (Ib) was facilitated by the clear demarcation between the radial hypertrophy zone and the old lamellar structure of the radial cortex, it was clear that osseous activity was still continuing in the control group, which led to an under-estimation of the true power of our engineered periosteal substitute.

In modifying the rabbit ulna defect, we combined the principles of tissue engineering with the advantages of GBR. Placing a non-resorbable GORE-TEX membrane around the radius, allowed an accurate evaluation of the contribution of the tissue-engineered periosteal substitute to bone regeneration where it resulted in a threefold increase in the amount of bone present in the defect as compared to the control group. Indeed, a true control was only possible when the GORE-TEX membrane was used. Furthermore, the presence of the barrier membrane permitted a clear demarcation of the original defect location and allowed an unbiased estimation of the amount of bone regenerated via both μCT and histological analyses.

One limitation of the study is the lack of complete defect bridging in the defects that received the periosteal substitute. This may be due to the fact that in such a critical size defect, the role of the cells in the periosteal substitute alone is not enough and that a cellular component within the scaffold itself is also required. Another drawback is the small number of animals per group, which did not allow a statistical analysis, Nevertheless, by this pilot study, we obtained clear evidence that modifying the rabbit ulnar defect model by adopting an intervening non-resorbable barrier membrane greatly enhanced the validity of the model for objective evaluation of the bone formation induced by the engineered periosteal substitute.

## Conclusion

We modified a rabbit ulnar defect model using a GORE-TEX barrier membrane around the radius to receive a periosteal substitute composed of a PRP/BMSC membrane around a poly (ester urethane) scaffold. The barrier membrane induced no immunoreactivity, was easy to apply, completely isolated the defect, and remained cell occlusive and intact. It allowed a clear demarcation of the defect area eliminating any interference from the surrounding tissues and allowing an unbiased objective interpretation of the results. On the contrary to the use of the commonly adopted rabbit ulna defect model, when using the barrier membrane, we could show that the PRP/BMSC periosteal substitute induced a threefold increase in the amount of bone regenerated as compared to the control.

## Conflict of Interest Statement

The authors declare that the research was conducted in the absence of any commercial or financial relationships that could be construed as a potential conflict of interest.
